# Exploring the Mental Health Challenges of Emergency Medicine and Critical Care Professionals: A Comprehensive Review and Meta-Analysis

**DOI:** 10.7759/cureus.41447

**Published:** 2023-07-06

**Authors:** Tarek Ibrahim, Amr Gebril, Mohammed K Nasr, Abdul Samad, Hany A Zaki

**Affiliations:** 1 Emergency, NMC Specialty Hospital, Al Ain, ARE; 2 Emergency Medicine, NMC Royal Hospital, Khalifa City, ARE; 3 Emergency Medicine, Dr. Sulaiman Al Habib Hospital, Dubai, ARE; 4 Acute Medicine/Emergency, NMC Royal Hospital, Khalifa City, ARE; 5 Emergency Medicine, Hamad Medical Corporation, Doha, QAT

**Keywords:** intensive care unit, systematic review, meta-analysis, critical care medicine, emergency medicine, mental health, burnout, depression

## Abstract

Burnout and depression are global problems affecting healthcare providers, especially those working in stressful departments such as emergency departments (EDs) and critical care units (CCUs). However, pooled data analysis comparing healthcare providers operating in the ED and CCU is yet to be conducted. Therefore, this meta-analysis was systematically conducted to investigate and compare the prevalence of burnout and depression among emergency medicine (EM) and critical care medicine (CCM) professionals.

We systematically searched for articles related to our research topic using the database search method and manual search method, which involved reviewing the reference lists of articles from electronic databases for additional studies. After screening the literature from the databases using the eligibility criteria, a quality appraisal using the Newcastle-Ottawa scale was performed on the eligible studies. In addition, a meta-analysis using the Review Manager software was performed to investigate the prevalence rates of burnout and depression.

A total of 10 studies with 1,353 EM and 1,250 CCM professionals were included for analysis in the present study. The pooled analysis did not establish any considerable differences between EM and CCM healthcare workers on the prevalence of high emotional exhaustion (EE) (odds ratio (OR) = 1.01; 95% confidence interval (CI) = 0.46-2.19; p = 0.98), high depersonalization (OR = 1.16; 95% CI = 0.61-2.21; p = 0.64), low personal accomplishment (PA) (OR = 0.87; 95% CI = 0.67 - 1.12; p = 0.28), and depression (OR = 1.20; 95% CI = 0.74-1.95; p = 0.45). Moreover, pooled data showed no considerable differences in EE scores (mean difference (MD) = -1.07; 95% CI = -4.24-2.09; p = 0.51) and depersonalization scores (MD = -0.31; 95% CI = -1.35-0.73; p = 0.56). However, EM healthcare workers seemed to have considerably lower PA scores than their CCM counterparts (MD = 0.12; 95% CI = 0.08-0.16; p < 0.00001).

No considerable difference was recorded in the prevalence of burnout and depression between EM and CCM healthcare workers. However, our findings suggest that EM professionals have lower PA scores than CCM professionals; therefore, more attention should be paid to the mental health of EM professionals to improve their PA.

## Introduction and background

Burnout and depression are significant concerns among healthcare providers worldwide. Burnout is described as a multifaceted syndrome characterized by high emotional exhaustion (EE), high depersonalization, and low sense of personal accomplishment (PA) [1,2]. High EE describes healthcare providers who feel exhausted, tired, depleted, and have a loss of energy. At the same time, depersonalization refers to those with a negative or inappropriate attitude toward their patients, irritation, and withdrawal. On the other hand, low PA refers to a reduction in productivity and incapacity to cope [3]. Research has shown that burnout in healthcare workers is usually related to several work-related and non-work-related factors. Some of the work-related factors associated with high burnout among healthcare workers include the number of working hours, work-related stress, high workload [4,5], working shifts [6], and lack of support from organizations [7]. Conversely, non-work-related factors include individual dispositions such as personality traits that influence job stress experiences.

On the other hand, depression is a major dissociative disorder characterized by persistent sadness and loss of interest and can influence one’s behavior and thinking capability. Research has shown that depression prevalence among healthcare workers ranges from 21.53% to 32.7% in high-income nations and is much higher than that of the general population worldwide [8-10]. It is also worth noting that both burnout and depression among healthcare workers have negative repercussions on the healthcare system. These repercussions include patient dissatisfaction, high turnover rates, medical errors, and associated financial costs [7,11-13]. Therefore, understanding the prevalence of burnout and depression among healthcare workers, especially in stressful environments such as emergency departments (EDs) and critical care units (CCU), is an excellent step toward managing these conditions.

Although extensive research on burnout and depression among healthcare workers has been carried out, no systematic review has been conducted to assess burnout and depression among healthcare workers in emergency medicine (EM) and critical care medicine (CCM). Therefore, this study directly compared the prevalence of these conditions in EM and CCM healthcare professionals.

## Review

Methodology

Eligibility Criteria

The set of conditions used to include or exclude articles for review in this study was derived from one experienced reviewer. Articles were mandated to satisfy the following requirements to be included in our review: randomized trials or observational studies written and published in the English language (this criterion eliminated direct translations of scientific terms that would otherwise influence our scientific research); studies that directly compared either depression or burnout or both between EM and CCM healthcare workers; studies that had sufficient sample sizes, i.e., more than 50 participants (this criterion helped us to improve the statistical power of our meta-analyses); studies whose results were either prevalence or mean scores of depression and burnout; and studies that used the three domains of the Maslach Burnout Inventory (MBI) scale (i.e., EE, depersonalization, and PA) to investigate the prevalence of burnout.

Conversely, studies were considered ineligible for review due to the following reasons: studies designed as either abstract without full articles, systematic reviews and meta-analyses, letters to the editor, case reports or case series, and studies individually investigating the prevalence of depression or burnout in either EM or CCM professionals.

Literature Search

Articles relevant to our research were searched according to the Preferred Reporting Items for Systematic Reviews and Meta-Analyses (PRISMA) guidelines. During the search, we employed two methods, including a database search using predefined keywords and the Boolean expressions “AND” and “OR” in five electronic databases (PubMed, ScienceDirect, Medline, Google Scholar, and Scopus) and a manual search which involved identifying additional studies by reviewing the reference lists of the articles from these electronic databases. The keywords used in the search were as follows: (“Burnout” OR “Burnout syndrome”) AND (“Depression” OR “Depressive symptoms” OR “mental health”) AND (“emergency units” OR “emergency medicine” OR “emergency department”) AND (“critical care unit” OR “critical care” OR “critical care medicine” OR “intensive care unit” OR “ICU”) AND (“Healthcare workers” OR “Healthcare professionals” OR “Healthcare providers” OR “physicians” OR “nurses”). In our search, we avoided all close or exact duplicates and gray literature to improve our scientific research. Moreover, studies were not limited to a specific duration of publication.

Data Extraction

The process of retrieving data from included studies was performed by two experienced reviewers who then summarized the data in a characteristic table. The data retrieved comprised author ID (surname of the first author and year of publication), study design, location (country) in which the study was performed, characteristics of the participants (sample size, profession, gender distribution, and mean/median age), measures of burnout and depression, and main outcomes. The primary endpoint of our study was the prevalence and magnitude of burnout and depression. The magnitude of burnout was analyzed by comparing the mean scores of EE, depersonalization, and PA. During the data extraction process, inconsistencies were resolved through a constructive dialogue between the two reviewers or by including a third reviewer who acted as an arbiter.

Quality Appraisal

All studies used for quantitative and qualitative analysis in this study were designed as observational, non-randomized studies; therefore, the two reviewers tasked with quality assessment agreed to use the Newcastle-Ottawa scale. This appraisal tool is usually categorized into three domains, namely, selection, comparability, and outcomes. Four assessment criteria are used under the selection domain, while two assessment criteria are employed in the comparability domain. On the other hand, the outcome domain usually consists of three assessment criteria. During the appraisal process, the reviewers assigned ratings of “1” or “0” for fully addressed and unclear or not addressed responses, respectively. Finally, the quality of each study was categorized into good, fair, or poor quality based on the conversion to the Agency of Healthcare Research and Quality standards. According to these standards, sound quality is used when the selection domain has a rating of either 3 or 4, comparability has a rating of 1 or 2, and the outcome domain has a rating of 2 or 3. Moreover, fair quality is used for rating scores of 2 in the selection domain, 1 or 2 in the comparability domain, and 2 or 3 in the outcome domain, while poor quality is used for scores of 0 or 1 in the selection domain, 0 in the comparability domain, and 0 or 1 in the outcome domain.

Data Synthesis

The overall effect sizes and statistical differences between the prevalence and magnitude of burnout and depression were calculated using the Review Manager software (RevMan 5.4.1). Data on the prevalence of burnout and depression was dichotomous; therefore, a meta-analysis was performed using the simple odds ratio (OR). Conversely, data regarding the magnitude of burnout was continuous; thus, calculations were made using the mean difference (MD). We also employed a random-effect model to counter heterogeneity and a 95% confidence interval (CI) for all calculations. The heterogeneity was calculated using I^2^ statistics, wherein scores of 0-49, 50-69, and 70-100 were considered low, moderate, and high, respectively. The final results were then presented as forest plots with significance levels of p < 0.05 deemed significant or considerable.

Results

Study Selection

**Figure 1:**
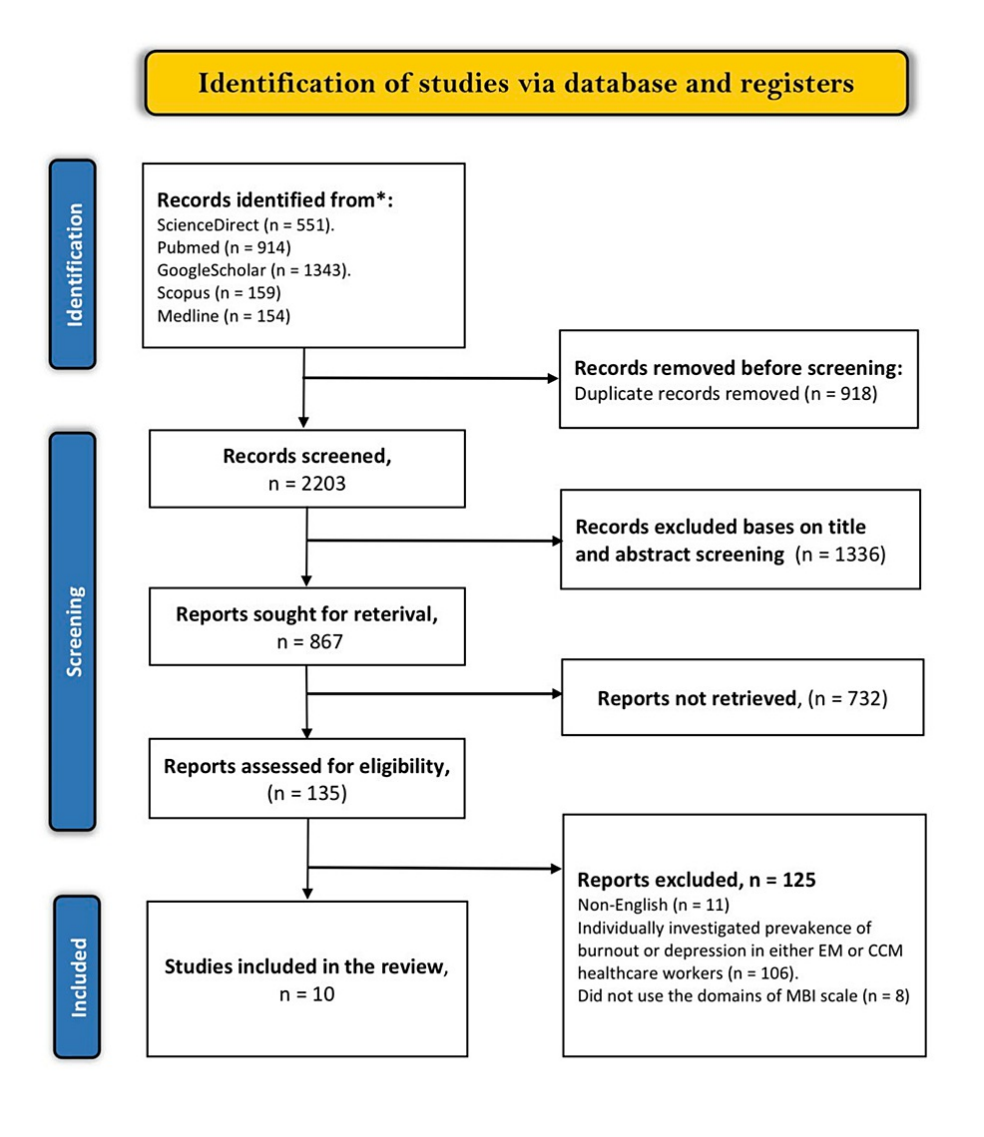
PRISMA flow diagram of the search results. PRISMA = Preferred Reporting Items for Systematic Reviews and Meta-Analyses

The electronic database search method yielded 3,121 articles relevant to the specified keywords. A duplicate analysis of these articles led to the exclusion of 918 articles deemed either close or exact duplicates. The abstracts and titles of the remaining articles were then screened, and 1,336 articles that did not meet the screening criteria were excluded. Out of the 867 remaining articles, 732 were not retrieved because they were either systematic reviews, literature reviews, letters to the editor, abstracts without full articles, or questionnaires. Finally, we only included 10 studies for review and analysis as the other 125 were excluded due to the following reasons: 11 were published in other languages, 106 individually evaluated either burnout or depression in either EM or CCM healthcare workers, and eight did not use the three domains of the MBI scale to assess burnout prevalence. The PRISMA flow diagram below summarizes the full study selection criteria (Figure 1). The study characteristics are presented in Table 1.

**Table 1:** Study characteristics. EE = emotional exhaustion; PA = personal accomplishment; ICU = intensive care unit; CCU = critical care unit; PICU = pediatric intensive care unit; ER = emergency room; ED = emergency department; PED = pediatric emergency department; MBI = Maslach Burnout Inventory; MBI-HSS = Maslach Burnout Inventory Human services Survey; MBI-GS = Maslach Burnout Inventory General Survey; BDI = Beck’s Depression Inventory; NR = not reported; SDS = Self-reporting Depression Scale; STAI = State-Trait Inventory; PHQ = Patient Health Questionnaire

Author ID	Study design	Location	Participants’ characteristics	Measures		Main outcomes
				Burnout	Depression	
Paes et al. (2022) [14]	An exploratory and descriptive study with a quantitative approach	Brazil	53 nursing professionals	MBI	NR	31.36% of nursing professionals in the ER had high EE, 30.92% had low professional fulfillment, and 39.25% had high depersonalization. 36.36% of nursing professionals in the ICU had high EE, 36.36% showed low professional fulfillment, and 22.73% had high depersonalization
Lozano et al. (2020) [15]	An observational, cross-sectional study	Spain	90 health professionals (47 nurses, 19 physicians, and 24 nursing aides; 25 males and 65 females; mean age: 42 ± 7.40 years)	MBI-HSS	NR	There was no difference in EE, depersonalization, and PA scores between the emergency unit and CCU health professionals (p = 0.24, p = 0.25, and p = 0.87, respectively).
Lasalvia et al. (2021) [16]	Longitudinal observational study.	Italy	1,961 health workers (286 physicians, 335 residents, 687 nurses, and 466 other healthcare staff; 492 males and 1,463 females	MBI-GS	NR	104 professionals working in the ICU had a high EE, 87 had low professional efficacy, and 73 showed high cynicism. 57 professionals working in the ED presented high EE, 64 showed low professional efficacy, and 42 had high cynicism
Rocha et al. (2019) [17]	Cross-sectional study	Brazil	91 health professionals (53 females and 38 males); 34 nursing technicians, 22 nurses, 12 physical therapists, and 23 physicians; mean age: 37 years	MBI-HSS	NR	Professionals in the ED had a significantly lower EE than ICU professionals (2.54 ± 0.09 vs. 3.00 ± 0.12; p < 0.001). No difference was observed between professionals in the ED and ICU on the intensity of low PA (3.78 ± 0.07 vs. 3.66 ± 0.10, respectively; p = 0.43) or depersonalization (1.93 ± 0.09 vs. 2.08 ± 0.12; respectively; p = 0.31)
Yacizi et al. (2019) [18]	Observational, cross-sectional, multicenter study	Turkey	570 health professionals (354 females and 216 males); 19 interns, 89 pediatric residents, 25 fellows of PICU or PED, 27 PICU or emergency specialists, and 269 nurses	MBI	NR	The prevalence of high EE was significantly higher in professionals working in the ED than ICU (67.7% vs. 59.9%. p = 0.015, respectively). No difference was observed in the prevalence of either depersonalization or low personal achievement between professionals working in the ED or ICU (p = 0.075 and p = 0.608, respectively). Generally, burnout syndrome was significantly higher in professionals working in the ED as opposed to those working in the ICU (79.1% vs. 73.7%, respectively; p = 0.04).
Vadi et al. (2022) [19]	Cross-sectional study	India	153 healthcare professionals (64 males and 89 females)	NR	BDI	58.3% of health professionals working in the ER had normal ups and downs, 33.3% had mild mood disturbance to borderline depression, and 8.4% had moderate to extreme depression. 54.3% of healthcare professionals working in the ICU presented normal ups and downs, 23.8% showed a mild mood disturbance to borderline clinical depression, and 21.9% had moderate to extreme depression
Habadi et al. (2018) [20]	Cross-sectional study	Saudi Arabia	182 nursing staff (17 males and 165 females)	MBI-HSS	NR	Of the 17 nursing staff working in the ED, 14 showed high EE, 5 presented high depersonalization, and 3 had a low PA. Out of 46 nursing staff working in the ICUs, high EE was recorded in 22 staff, high depersonalization in 11 staff, and low PA in 11 staff
Alanazi et al. (2020) [21]	Cross-sectional study	Saudi Arabia	3,557 healthcare professionals (2,063 males and 1,494 females); 823 doctors, 1,361 nurses, 467 technicians, and 906 with other specialties	MBI	NR	The 606 healthcare professionals in the ED showed a mean EE of 23.54 (16.09), mean depersonalization of 7.96 (7.69) and mean PA of 33.94 (10.98). The 184 healthcare professionals in the ICUs showed a mean EE of 27.96 (16.04), a mean depersonalization of 7 9.30 (7.32), and a mean PA of 33.10 (9.54)
Al Mutair et al. (2021) [22]	Descriptive, cross-sectional, non-interventional study.	Saudi Arabia	642 healthcare professionals (167 males and 475 females); 114 physicians, 323 nurses, and 207 with other specialties	NR	Zung SDS	Of the 52 healthcare professionals working in the ED, 35 had normal depression, 13 had mild depression, 3 had moderate depression, and 1 had severe depression. Of the 243 healthcare professionals working in the ED, 173 had normal depression, 58 had mild depression, 8 had moderate depression, and 4 had severe depression
Tabur et al. (2022) [23]	Cross-sectional survey study	Turkey	344 healthcare providers (55 males and 289 females)	STAI-I/STAI-II	PHQ-9	Of the 145 healthcare professionals working in the ER, 41.80% had mild or low depression, 43.55% had moderate depression, and 40.74% had high or severe depression. Of the 25 healthcare professionals working in the ER, 3.28% had mild or low depression, 12.90% had moderate depression, and 9.26% had high or severe depression

Quality Appraisal Results

The quality assessment showed that six studies had a fair methodological quality while four had good methodological quality (Table 2).

**Table 2:** Methodological quality using the Newcastle-Ottawa scale.

Author ID	Selection (maximum 4)	Comparability (maximum 2)	Outcome (maximum 3)	Quality
Paes et al. (2022) [14]	2	1	2	Fair
Lozano et al. (2020) [15]	3	2	2	Good
Lasalvia et al. (2021) [16]	2	2	2	Fair
Rocha et al. (2019) [17]	4	2	2	Good
Yacizi et al. (2019) [18]	3	2	1	Good
Vadi et al. (2022) [19]	2	2	2	Fair
Habadi et al. (2018) [20]	3	2	2	Good
Alanazi et al. (2020) [21]	2	2	2	Fair
Al Mutair et al. (2021) [22]	2	2	2	Fair
Tabur et al. (2022) [23]	2	2	2	Fair

In most cases, the outcome assessment results were influenced by the fact that all studies used self-reporting measures for burnout and depression, implying that responder bias was introduced in our systematic review.

Burnout

**Figure 2:**
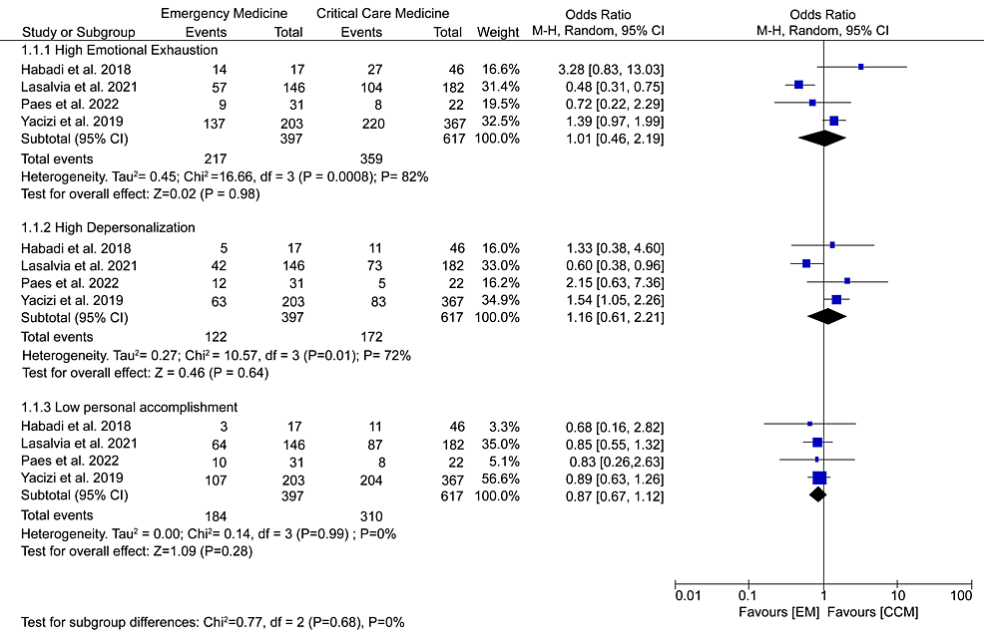
A forest plot comparing the prevalence of burnout among EM and CCM healthcare professionals. Paes et al. (2022) [14], Lasalvia et al. (2021) [16], Yacizi et al. (2019) [18], Habadi et al. (2018) [20]. EM = emergency department; CCM = critical care medicine

As shown in our summary of study characteristics (Table 1), the MBI scale was the most commonly used measure for burnout. Therefore, a subgroup analysis of high EE, high depersonalization, and low PA was used to compare the prevalence of burnout between EM and CCM healthcare professionals. Data pooled from four studies did not demonstrate any considerable difference between EM and CCM healthcare workers on the prevalence of high EE (OR = 1.01; 95% CI = 0.46-2.19; p = 0.98), high depersonalization (OR = 1.16; 95% CI = 0.61-2.21; p = 0.64), and low PA (OR = 0.87; 95% CI = 0.67-1.12; p = 0.28) (Figure 2).

**Figure 3:**
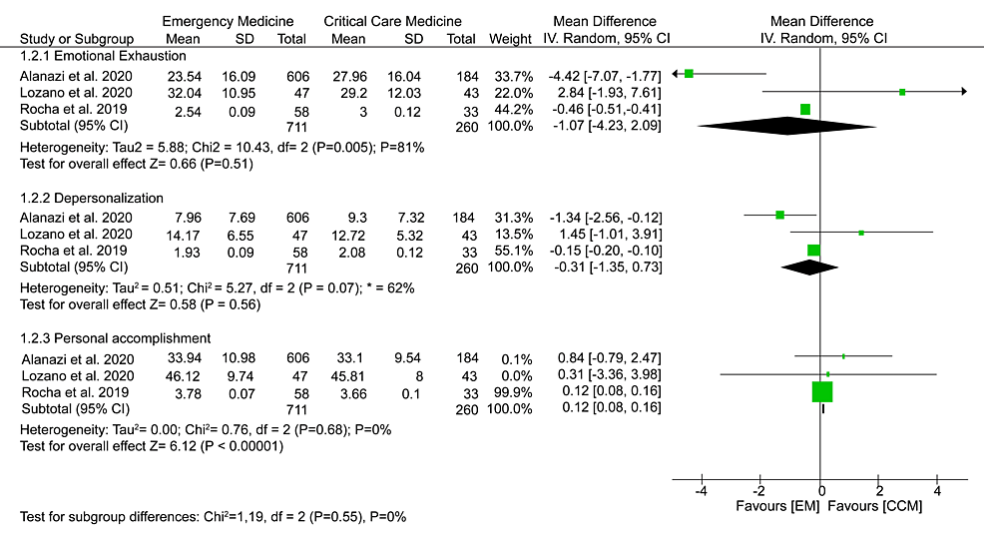
A forest plot comparing the magnitude of burnout among EM and CCM healthcare professionals. Lozano et al. (2020) [15], Rocha et al. (2019) [17], Alanazi et al. (2020) [21]. EM = emergency department; CCM = critical care medicine

Moreover, no significant difference was recorded in EE scores (MD = -1.07; 95% CI = -4.24-2.09; p = 0.51) and depersonalization scores (MD = -0.31; 95% CI = -1.35-0.73; p = 0.56). However, EM healthcare workers seemed to have considerably lower PA scores than their CCM counterparts (MD = 0.12; 95% CI = 0.08-0.16; p < 0.00001) (Figure 3).

Depression

**Figure 4:**
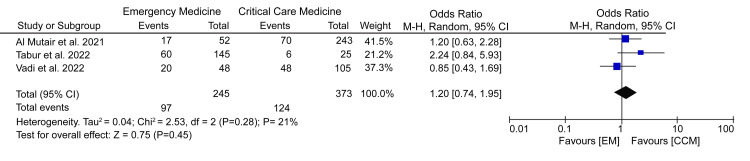
A forest plot comparing the prevalence of depression among EM and CCM healthcare professionals. Vadi et al. (2022) [19], Al Mutair et al. (2021) [22], Tabur et al. (2022) [23]. EM = emergency department; CCM = critical care medicine

From our summarized study characteristics, it is evident that three different scales were used to quantize depression in EM and CCM health professionals. Therefore, we performed a meta-analysis on the overall prevalence of depression rather than a subgroup analysis of different levels of depression, which included low, mild, moderate, severe, or extremely severe. Data pooled from the three studies reporting the prevalence of depression showed no considerable difference between EM and CCM professionals (OR = 1.20; 95% CI = 0.74-1.95; p = 0.45) (Figure 4).

Discussion

EDs and CCUs are some of the most stressful departments in hospitals, and, as such, healthcare workers in these departments are at a greater risk of burnout and depression [24,25]. Therefore, this study was conducted to compare the prevalence and magnitude of burnout and depression among healthcare professionals in EM and CCM. Our analysis has shown no difference in the prevalence of depression, low PA, high depersonalization, and high EE between healthcare workers in EM and CCM. However, healthcare workers specializing in EM seem to have significantly lower PA scores than those specializing in CCM.

Our findings are consistent with previous studies showing no difference in the overall prevalence of burnout between EM and CCM health professionals. For example, a 2010 exploratory study with a cross-sectional design categorized burnout as either low, medium, or high and found no difference in the overall prevalence of burnout between nursing staff working in the ED and intensive care unit (ICU) (82% and 81% of nurses in ED and ICU reported moderate to high levels of burnout, respectively) [26]. Similarly, a study on nursing staff working in critical care settings in Rwanda reported an insignificant difference in the prevalence of burnout (p = 1.000) [27]. Moreover, our analysis has shown that significantly lower PA scores are observed among emergency personnel. This outcome was highly weighted by Rocha et al. [17], who claimed that PA scores were highly influenced by job satisfaction and the marital status of healthcare personnel.

Although we only compared the prevalence and magnitude of burnout between EM and CCM personnel, it is crucial to identify factors that increase the risk of burnout in this study population. The first factor associated with burnout is working shifts. In most professions, nights, weekends, and holidays are regarded as spare times reserved for rest and family; however, this is usually not the case for healthcare workers in EM and CCM. An observational cross-sectional study of professional burnout in physicians working in pediatric ICUs (PICUs) reported that physicians who were on-call for over 36 hours weekly were at a higher risk of burnout than those who were on-call for fewer hours (p = 0.03) [28]. Similarly, a French nationwide study of critical care nursing staff indicated that the frequency of monthly night shifts and the duration from the last non-working weeks were significantly associated with the increased risk for burnout [29]. On the other hand, a Saudi Arabian study investigating risk factors of burnout during the COVID-19 pandemic reported that healthcare personnel who worked for more than eight hours every day had higher incidences of high EE (OR = 1.48; 95% CI = 0.15-0.63; p = 0.001), high depersonalization (OR = 1.36; 95% CI = 0.08-0.53; p = 0.009), and low PA (OR = 1.36; 95% CI = 0.52-0.9; p = 0.004) [21]. Similarly, a cross-sectional study of 379 professionals working in EDs demonstrated that physicians having night shifts or day and night shifts had an increased incidence of EE (p = 0.022), depersonalization (p = 0.08), and low PA (p = 0.012) than those who worked during the day [30].

Demographic factors such as age, sex, and marital status have also been associated with an increased risk for burnout in our study population. Research has shown that young age is highly associated with an increased risk for burnout. For instance, a cross-sectional study of 93 nursing personnel working in the acute and critical care departments in Peru demonstrated that age increase was an independent factor for high depersonalization (p = 0.03) but not EE (p = 0.10) or PA (p = 0.38) [31]. On the other hand, Aytekin and colleagues demonstrated that nursing staff in the ICUs aged 36 years and older had a higher prevalence of low PA but not depersonalization or EE [32]. However, research by Merlani and colleagues specifically claimed that being younger than 40 years was a major risk for burnout in ICU caregivers [33]. A nationwide study of EM professionals also demonstrated a higher incidence of EE, depersonalization, and low PA among personnel aged below 45 years [34].

Furthermore, several studies have reported sex as a risk factor for burnout in EM and CCM professionals. Raggio and colleagues indicated that men, especially male doctors, had a higher incidence of depersonalization, while female physicians had a higher incidence of EE [35]. Similarly, a study of pediatric ED (PED) and PICU healthcare workers demonstrated that female professionals had a considerably higher incidence of EE than male professionals (70.9% vs. 49.1; p < 0.001). However, a Swiss study suggested that the female sex was associated with decreased risk of burnout [33]. Looking at these studies, it is evident that there is no consistency on which gender specifically increases the risk of burnout, meaning that gender risk is coupled with other risk factors to explain the observed variations. Research also suggests that being single or divorced and childless might be associated with an increased risk for burnout [33,36]. Furthermore, a study of PICU and PED health professionals demonstrated that being single or divorced and childless was significantly associated with high EE and depersonalization but not decreased PA.

There is active disagreement on the relationship between burnout and depression. Several studies have found that burnout is merely an atypical depressive syndrome with no distinguishing characteristics (i.e., burnout and depression overlap) [37]. This seems to be true as some burnout symptoms, such as loss of energy or fatigue, are similar to those of depression. However, Alwhaibi et al. [13] reported that depression was associated with an increased risk for EE, depersonalization, and personalization. This relationship is further reinforced by a previous systematic review of 67 studies [38], but most of the studies included in this review had a cross-sectional design, meaning that it is difficult to determine whether burnout leads to depression or vice versa. Therefore, more longitudinal studies are required to establish the relationship between burnout and depression fully.

It is also worth noting that burnout and depression in EM and CCM healthcare professionals negatively impact the healthcare system. Research suggests high burnout levels are associated with intentions to leave jobs, job turnovers, absenteeism, and decreased performance and productivity. A univariate regression analysis performed by Tabur and colleagues during the COVID-19 pandemic showed that physicians who had severe burnout levels were more likely to leave their jobs compared to those with low and moderate burnout levels (OR = 13.05; 95% CI = 1.10-33.48; p < 0.00001) [23]. However, the analysis found no relationship between moderate and severe depression levels with the intention to leave jobs. Another study of ICU nurses revealed an intention to leave jobs in about 50% of the nurses exhibiting high levels of burnout. Additionally, burnout has been associated with reduced quality and quantity of medical services offered by healthcare workers. Qiao and colleagues argued that when nurses are burned out, the quality and safety of services offered are affected, leading to increased errors in their work, reduced patient satisfaction, and increased patient mortality [39].

Moreover, there are reports that burnout and depression in healthcare professionals might affect their health and well-being. Suicidal thoughts are one of the major concerns of high burnout and depression among healthcare workers. A cross-sectional survey of 7,378 nurses working in various departments revealed that 5.5% of the nurses had experienced suicidal thoughts in the course of their work, which is higher than that of the general population (4.3%). Further analysis revealed that 38.2% of these nurses had at least one symptom of burnout, and 43.3% showed depression symptoms [40]. This finding is reinforced by a review of 20 published articles that reported nurses with high depression levels, low self-fulfillment, and burnout syndrome have a higher prevalence of suicides [41]. Depressed physicians/nurses usually feel they are failures, isolated, and cut off from their colleagues, who seem to cope better. It is due to these feelings that some of them are driven toward suicidal thoughts.

Although our analysis has shown no difference between EM and CCM healthcare workers, it is evident that the prevalence of burnout in each group is very high (54.7% and 58.2% EE, 30.7% and 27.9% depersonalization, and 46.3% and 50.2% PA in EM and CCM, respectively). Therefore, there is a need to effectively manage and prevent burnout in healthcare professionals. One intervention that has been found effective in managing burnout in health professionals is organizational strategies. These strategies include offering a dynamic and more flexible work schedule, improving working conditions, interchanging members of a particular team, and job rotations allowing health professionals to work in different units [33,42,43]. Working conditions can be improved by ensuring adequate staff, material, and good leadership in each department. Moreover, research has found individual-oriented strategies regarding practical and personal aspects effective in burnout management. Practical aspect strategies usually include providing education programs or seminars about the management of stress, communication skills sessions, and relaxation techniques such as yoga and mindfulness [44-46], while personal aspect strategies include career counseling, life coaching, and social, individual, and personality coping [47,48].

A study conducted among medical students revealed that sleeping was a significant predictor for burnout, with seven-hour sleep being adequate to ensure that the body had recovered from work exhaustion and regained its organic function [48]. Furthermore, it was reported that involvement in activities such as music and physical exercises reduced stress and burnout. Evidence also suggests burnout can be mitigated if healthcare workers balance life with their job. Research by Galinsky and colleagues revealed that people who spend equal time working and dealing with family issues are stress-free and are usually more productive in their work as opposed to those who mainly concentrate on work or family issues [49]. Moreover, the study revealed that taking breaks during jobs improved productivity. This can be explained by the fact that people who work continuously without breaks are more likely to experience burnout.

Limitations

The findings of this study should be interpreted while considering the following limitations; first, our study eligibility criteria were constructed to include articles published in the English language, meaning data that would have been used to improve our study findings were eliminated. Second, most studies included in this review were designed as cross-sectional studies, meaning that sampling was not random and the quality of evidence was mostly fair; therefore, causal relationships are difficult to draw from these studies. Furthermore, most of the data presented in these studies was self-reported, meaning that our conclusion may be biased due to the high risk of responder bias. Research has shown that many healthcare professionals are less likely to report depression as it remains stigmatized in medical culture; hence, incidences of depression might have been underreported, and, as such, the results of our meta-analysis may be biased. In addition, some of the studies included in this review were performed during the COVID-19 pandemic when psychological distress and stressful working conditions peaked. However, we did not differentiate data for studies during COVID-19 and the pre- or post-pandemic era which might prove to be a wrong decision if further studies comparing data in these eras are carried out. High heterogeneity in our meta-analyses has also been recorded. This heterogeneity can be attributed to the fact that sample sizes, locations of the study, and measuring scales varied between the studies. However, the heterogeneity did not influence our results because most of the studies had fair to good methodological quality. Finally, it is difficult to determine the exact risk factors of burnout and depression among EM and CCM healthcare professionals from our study due to limited data. Therefore, further studies that directly compare risk factors of burnout and depression among EM and CCM healthcare professionals will be vital in providing information that healthcare professionals and policymakers will use to tackle burnout and depression in this study population.

## Conclusions

In summary, our meta-analyses have shown that even though the prevalence of burnout and depression is high in emergency and critical care services, the difference in the two settings is statistically insignificant. However, our findings suggest that healthcare professionals specialized in EM have a significantly lower PA score than those specialized in CCM. Therefore, more focus should be paid to the mental health of EM healthcare professionals to improve their PA. Moreover, this review has discussed some risk factors that influence burnout in EM and CCM; however, they are still poorly understood, and more studies are warranted to establish their influence. Nevertheless, this systematic review has discussed the consequences of high burnout and depression levels on the healthcare system and the strategies employed to mitigate or manage these conditions. However, future studies designed as randomized trials must address the effective management and prevention strategies for burnout and depression among healthcare professionals specializing in EM and CCM.
